# Feasibility and acceptability of combining cognitive remediation and tDCS in long-term psychiatric clinical care^☆^

**DOI:** 10.1016/j.scog.2025.100358

**Published:** 2025-06-10

**Authors:** Anika Poppe, Leonie Bais, Daniëlle van Duin, Branislava Ćurčić-Blake, Gerdina Hendrika Maria Pijnenborg, Lisette van der Meer

**Affiliations:** a Department of Clinical and Developmental Neuropsychology, University of Groningen, Groningen, the Netherlands; b Department of Rehabilitation, Lentis Psychiatric Institute, Lagerhout E35, 9741 KE Zuidlaren, the Netherlands; c Phrenos Center of Expertise, Utrecht, the Netherlands; d Department of BSCS Neuroscience, University of Groningen, University Medical Center Groningen, Groningen, the Netherlands; e Department of Psychotic Disorders, GGZ Drenthe, Assen, the Netherlands

**Keywords:** tDCS, Cognitive training, Psychiatric rehabilitation, Severe mental illness, Psychosis, Schizophrenia

## Abstract

**Background and hypothesis:**

Cognitive impairments are commonly experienced by individuals with severe mental illness (SMI) and are associated with problems in everyday life. This pragmatic, randomized, controlled, pilot trial explored the acceptability, feasibility, and preliminary effects of cognitive remediation (CR) combined with transcranial direct current stimulation (tDCS) for cognitive and everyday functioning in individuals with SMI in long-term psychiatric clinical care. We hypothesized that combining CR and tDCS is feasible and acceptable to individuals with SMI.

**Study design:**

Twenty-four individuals with SMI were randomized to either CR + active tDCS (*n* = 13) or CR + sham tDCS (*n* = 11) over 32 sessions (16 weeks). Acceptability was evaluated in semi-structured interviews. Cognitive and everyday functioning were evaluated at baseline, post-16 week waiting period, post-intervention, and 6-months post-intervention.

**Study results:**

Overall, participants were positive about the training. Over 60 % of participants successfully finished at least 20 sessions, meeting the predefined criteria for feasibility. CR appeared to yield subjective improvements to participants, significant improvements in cognitive tests post-intervention and at follow-up and improved self-reported negative symptoms at follow-up. Observer-rated everyday functioning and cognition, and subjective cognitive complaints did not change following CR.

**Conclusions:**

This study concludes that CR is an acceptable and feasible intervention for individuals with SMI in long-term psychiatric clinical care. The addition of tDCS requires further investigation to ascertain its potential benefits.

## Highlights

• Combining cognitive remediation and tDCS is feasible.
• The treatment combination is acceptable to individuals with severe mental illness.
• Participants reported benefits in everyday life from cognitive remediation.
• tDCS did not add an insuperable burden to cognitive remediation.

## 1 Introduction

Severe mental illnesses (SMI), such as schizophrenia, bipolar disorder, and substance use disorder, are characterized by debilitating symptoms that can significantly affect everyday functioning (Parabiaghi et al., 2006; Delespaul and EPA de consensusgroep, 2013). A subset of individuals with SMI experiences severe and persistent cognitive impairments, negative symptoms, behavioral difficulties, and comorbid disorders, that substantially impact their ability to function independently, often requiring continuous and intensive psychiatric care and support (Wiersma et al., 1998; Killaspy, 2014; Trieman and Leff, 2002). While this group is heterogeneous in terms of specific diagnoses, cognitive impairments are a common and transdiagnostic feature (Abramovitch et al., 2021), serving as strong predictor of functional capacity, social skills, and everyday functioning (Kurtz, 2006; Mahmood et al., 2018).

Cognitive remediation (CR) is an umbrella term for interventions aiming to improve cognitive processes with the goal of durability and generalization to everyday life (Cognitive Remediation Expert Workshop 2023). Across the board, meta-analyses have shown the effectiveness of CR in improving everyday and cognitive functioning for individuals with psychotic disorders with moderate to large effects (Wykes et al., 2011; Vita et al., 2021). The observed effects are larger for CR interventions including four core elements (Vita et al., 2021), which were identified by cognitive remediation experts (Bowie et al., 2021): A trained and active therapist, problem-solving strategies, generalization procedures, and cognitive exercises (Bowie et al., 2021). Given that cognitive impairments are not exclusive to psychotic disorders but are prevalent across the SMI spectrum and are considered a transdiagnostic symptom (Abramovitch et al., 2021), CR represents a promising intervention for a broad range of individuals with SMI, provided they exhibit cognitive impairments.

Previous studies have primarily focused on service users receiving outpatient care leaving a gap in understanding the impact of CR on individuals with SMI in long-term intensive psychiatric clinical care. These individuals often exhibit the highest level of cognitive impairments and the lowest level of functioning (Killaspy, 2014). For instance, in a meta-analysis that assessed the effectiveness of CR in inpatients with psychosis five out of the 20 included studies were conducted in long-term clinical cares settings (Cella et al., 2020). The meta-analysis showed that CR is effective and acceptable for improving cognitive outcomes in inpatient settings, but improvements in everyday functioning were inconsistent. This may be due to the limited opportunities in an inpatient setting to perform activities related to the goals people have in everyday life, to perform activities that are assessed with functional outcome measure, and differences in CR program designs. Only five studies, including two in long-term care, included all core elements, which might explain the inconsistent functional improvements.

The four core elements are one, but not the only, approach that has been studied to enhance the potential benefits of CR. Non-invasive brain stimulation techniques as transcranial direct current stimulation (tDCS), which modulate neural activity, promise significant cognitive and functional improvements with shorter training (Jahshan et al., 2017). As individuals with SMI exhibit abnormal neural plasticity affecting cognition (Stephan et al., 2009; Beneyto and Meador-Woodruff, 2008), tDCS could enhance cognitive performance. The underlying working mechanism of tDCS is based on the administered current altering the excitability of neurons, thereby modulating spontaneous neuronal network activity through a tDCS polarity-dependent shift of the resting membrane potential (Paulus, 2011). When combined with CR, it is hypothesized that tDCS can enhance the activation of target regions involved in the processes (Guleyupoglu et al., 2013). However, research on this combination is limited. In a transdiagnostic meta-analysis focusing on the combination of cognitive training interventions and non-invasive brain stimulation (Poppe et al., 2023), we demonstrated that many studies do not fully address everyday functioning in the training and, thus, do not meet the criteria for CR classification (Bowie et al., 2021). Other studies did not adequately measure improvements in everyday functioning nor focused on people with SMI. Moreover, no studies evaluated the efficacy of combining cognitive training interventions and tDCS in psychiatric inpatient settings. Hence, the feasibility, acceptability, and potential benefits of combining CR with tDCS for enhancing cognitive and everyday functioning in individuals with SMI remain unclear, necessitating further research to evaluate its applicability in clinical settings.

The main aim of this pragmatic, randomized, controlled pilot trial is to investigate feasibility and acceptability of (1) using a CR program that incorporates the four core elements in long-term psychiatric clinical care for individuals with SMI, and (2) integrating tDCS with CR. Participants received CR either combined with active or sham tDCS after a 16-week waiting period, allowing for insights from a within- and between-subjects perspective. This study holds the potential to expand our understanding of the acceptability, feasibility, and clinical relevance of CR in long-term psychiatric clinical care for individuals with SMI and explores the feasibility of augmenting its outcomes with tDCS.

## 2 Methods

### 2.1 Study design

**Fig. 1:**
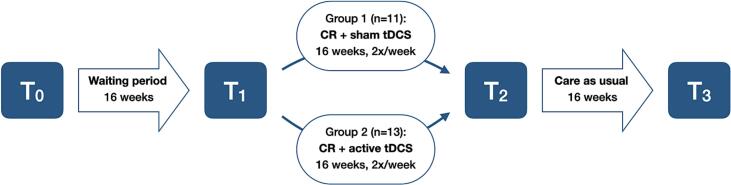
Study design.

This trial was a pragmatic, randomized, controlled, pilot trial following a non-concurrent multiple baseline design investigating the combination of CR and tDCS in people with SMI (see Fig. 1 for an overview of the study design). This study was approved by the Medical Ethics Review Committee of the University of Groningen (METc2018/555) and prospectively registered in the Dutch Trial Register (NTR7954). The study protocol was published (Poppe et al., 2021), and the code and full results are available in the open science framework (https://osf.io/cj49e/).

### 2.2 Participants

Eligible participants were individuals with a severe mental illness—a psychiatric disorder requiring treatment with no remission of symptoms, severe disabilities in social and societal functioning for at least two years which are the result of a psychiatric disorder, and needing coordinated professional care (Parabiaghi et al., 2006; Delespaul and EPA de consensusgroep, 2013)—who lived in a long-term psychiatric hospital or sheltered living facility of Lentis Psychiatric Institute (Northern Netherlands). These settings are characterized by the 24/7 availability of mental health care workers who support the service users with their everyday living activities (for more information, see (Zomer et al., 2020)). Other inclusion criteria were: (1) aged at least 18 years, (2) sufficient mastery of the Dutch language, and (3) having cognitive impairments (assessed in the baseline measurement).

Exclusion criteria were having previously received CIRCuiTS or contraindications for tDCS (metal or electronic implants inside the skull or eye, severe scalp skin lesions, pregnancy, and/or a history of previous seizures). All participants gave written informed consent and were compensated for completing assessments (10 euro per assessment) and the interview (5 euro). The recruitment procedure is described in the supplements.

### 2.3 Randomization and blinding

After completing T1, participants were randomized in a 1:1 ratio using permuted blocks of random sizes to either CR + active tDCS or CR + sham tDCS by an independent researcher using a randomization plan generator. The allocation sequence was sealed in an envelope until the data collection was completed. The researchers, therapists, and participants were kept blind to the tDCS condition. The outcome assessors (trained psychology undergraduates) were kept blind to the design and aims of the study confirmed by post-assessment questionnaires (see supplements for more information on randomization and blinding).

### 2.4 Interventions

The combined intervention was provided twice weekly for 16 weeks (32 sessions). Sessions lasted 20–45 min depending on the participant's attention span. tDCS was applied by the therapist concurrently with CR during the first 20 min of the session. Missed sessions were not replaced. All participants received treatment as usual during the whole study procedure, which consisted of a combination of therapies (e.g., cognitive behavioral therapy, creative therapy, psychomotor therapy) and activities.

#### 2.4.1 Cognitive remediation

We used the computerized CR program CIRCuiTS (Reeder et al., 2017; Reeder et al., 2016), which includes the four core elements of CR as defined by Bowie et al. (2021). This program focusses on cognitive practice, strategy use, and meta-cognitive engagement. The cognitive tasks (attention, visual and verbal memory, and planning) progress from abstract to more complex ecologically valid tasks. Problem-solving strategies are suggested by the program and therapist, tracked, and evaluated within the program, and discussed between participants and the therapist. The therapist encourages participants to regulate and monitor their cognitive performance within the training to encourage meta-cognitive engagement and discusses the transfer of these trained skills to everyday life. The therapy plan is adapted to the participants' personal goals and performance.

The therapists were two trained graduate-level psychologists (AP and a graduated and experienced psychologist) who followed the online CIRCuiTS training program (https://www.circuitstherapyinfo.com/training). They received monthly supervision by two experienced cognitive remediation therapists (GP and DD).

#### 2.4.2 Transcranial direct current stimulation

We administered tDCS with an Eldith DC stimulator (NeuroConn). The target area was the left dorsolateral prefrontal cortex (DLPFC) by placing the anode on C3 and cathode on Fp1. The electrode locations were determined with computational modelling using the SimNIBS software (see supplements) (Saturnino et al., 2019). The electrodes were fixed with conductive paste. For active tDCS, a current of 2 mA was administered for 20 min with a fade in/out of 30 s. We limited stimulation to the first 20 min of training because evidence suggests that longer stimulation might reduce its effectiveness (Monte-Silva et al., 2013). For the sham tDCS, the fade in was identical to the active condition but followed by 30 s fade out. After that, a standard control pulse, with no therapeutic effect, was frequently sent to monitor electric conductivity.

### 2.5 Primary outcome measures: feasibility and acceptability

#### 2.5.1 Feasibility

Based on previous studies, we defined a priori (Poppe et al., 2021) that the intervention is feasible if more than 60 % of the sample completes the study (retention rate) (Cella et al., 2020; Reeder et al., 2017; Linke et al., 2019; Thomas and Rusten, 2019). Study completion was defined as attending at least 20 sessions of the intervention (participation rate) (Reeder et al., 2017; Reeder et al., 2016).

#### 2.5.2 Acceptability

The intervention acceptability was assessed through semi-structured interviews (Table S2) conducted at participants' post-intervention assessment. Based on the Theoretical Framework of Acceptability (TFA) (Sekhon et al., 2018), we examined seven components of acceptability: Affective attitude, burden, intervention coherence, ethicality, opportunity costs, perceived effectiveness, and self-efficacy. The semi-structured interview included questions and example follow-up prompts for each TFA component (see Table S2). Seventeen of 24 participants agreed to participate in the interviews and provided written consent before the interview. Interviews were conducted by researchers (LB, LM) not involved in data collection or intervention delivery and either recorded and summarized or, if participants did not consent to recording, summarized directly after the interview. One researcher extracted and summarized all belief statements related to the different TFA components. To provide an overview of participant perspectives, we quantified how many participants made each statement (Table S5).

The feasibility and acceptability measures were assessed across the combined CR + tDCS group (including both active and sham tDCS conditions), as the procedural setup was identical for both groups. No significant differences in side effects were observed, and our manipulation check confirmed successful blinding. For comprehensiveness, we report feasibility and acceptability for the separate groups in the results and supplements (Table S5).

### 2.6 Secondary outcome measures: preliminary effectiveness

The following measures were assessed at baseline 1 (before 16-week waiting period, T0), baseline 2 (pre-intervention, T1), post-intervention (T2), and 6-month post-intervention (T3):

• *Observer-rated Questionnaires.* Everyday functioning (Life Skills Profile, LSP) (Rosen et al., 1989) and observed cognitive functioning (Nurses Observation Scale for Cognitive Abilities, NOSCA) (Persoon et al., 2012) was rated by one of the case managers of the service user.
• *Subjective Questionnaires.* Self-reported cognitive functioning (Cognitive Failure Questionnaire, CFQ) (Broadbent et al., 1982; Ponds et al., 2006) and self-reported negative symptoms (Self-report of Negative Symptoms, SNS) (Dollfus et al., 2019).
• *Neurocognitive measures.* Processing speed (Controlled Oral Word Association Test, COWAT) (Schmand et al., 2008), attention (WAIS-IV Digit Span Forward) (Wechsler, 2008), working memory (WAIS-IV Digit Span Backward) (Wechsler, 2008), visual memory (Rey Complex Figure Text) (Rey, 1941; Osterrieth, 1944), verbal memory (15-word learning task, 15-WT) (Saan and Deelman, 1986), and reasoning and problem solving (Stroop Color and Word Test; Modified Card Sorting Test, MCST) (Nelson, 1976; Stroop, 1935) were assessed.

For the LSP, NOSCA, and global cognition (standardized), a higher score represents a more favorable outcome. For the CFQ and SNS, a lower score represents a more favorable outcome.

#### 2.6.1 Baseline measures

Demographical information (age, sex, level of education, primary diagnosis, illness duration, age of onset, chlorpromazine equivalent), Positive and Negative Syndrome Scale (PANSS) (Kay et al., 1987) scores, motivational reasons to participate, and expectations regarding training effectiveness were collected at baseline.

More information on the outcome and baseline measures is available in the supplements.

### 2.7 Statistical analysis

All analyses were conducted on intention-to-treat samples using multilevel models in R (Version 4.3.0) with the ‘lme4’ package. Linear mixed models predicted each outcome measure (e.g., LSP mean score) from assessment time, with T1 as the reference level to compare T1 with T0 (16-week waiting period), T1 with T2 (post-treatment), and T1 with T3 (6-month follow-up). Three models were built for each outcome: (1) random intercepts, (2) random slopes added, and (3) tDCS (active vs. sham) added as a predictor. Non-significant T1 vs. T0 effects were excluded from the final model. Fixed effects' significance was evaluated using t-values, and beta coefficients indicated the expected change in outcomes between assessment times. Negative values for T1 vs. T0 indicate score increases over time, while positive values for T1 vs. T2 and T1 vs. T3 indicate score increases.

## 3 Results

### 3.1 Participants

**Fig. 2:**
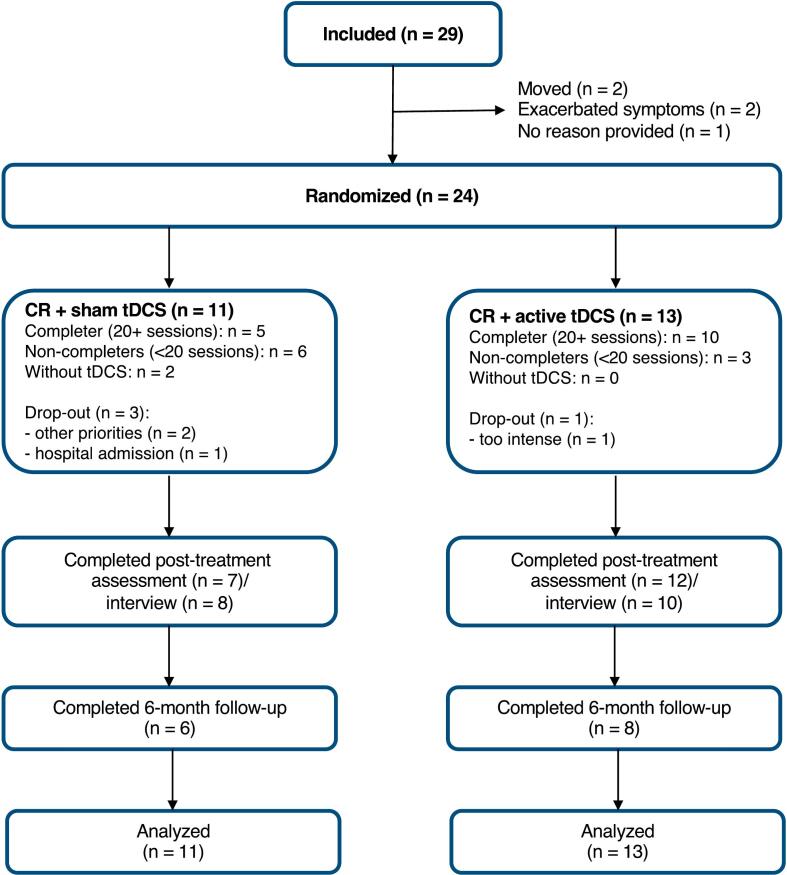
Study flow diagram. Note. Due to the individualized recruitment strategies employed across departments, consistent data on the initial number of individuals screened for our trial was not obtainable. The diversity in recruitment methods, ranging from personal outreach to broad departmental presentations, led to substantial variability that cannot be accurately quantified or compared. Consequently, we have omitted these figures from our report as they do not reflect a standardized measure of initial participant engagement.

In total, 29 participants from seven different departments were included between October 2019 and April 2022; five participants dropped out before randomization. The remaining 24 participants were randomized to sham tDCS + CR (*n* = 11) and active tDCS (*n* = 13). Two participants in the sham tDCS + CR condition did not receive tDCS (skin reaction to electrodes and fear of having a skin reaction to electrode paste in first session). Fig. 2 shows the participant flow diagram. Table 1 summarizes the demographics and clinical characteristics.

**Table 1:** Participant demographics and clinical characteristics.

Baseline characteristic	CR + sham tDCS (*n* = 11)	CR + active tDCS (*n* = 13)	*p*
Age	45.4 (16.1)	33.1 (12.6)	0.053
Female	6 (54.5 %)	3 (23.1 %)	0.245
Level of education^a^			0.244
Low	3	2	
Middle	5	3	
High	3	8	
Primary diagnosis^b^			0.407
Schizophrenia	2	5	
Autism spectrum disorder	3	1	
Schizoaffective disorder	1	2	
Bipolar disorder	1	1	
Major depressive disorder	2	0	
Psychosis NOS	0	1	
Borderline personality disorder	1	0	
PTSD	0	1	
Body dysmorphic disorder	0	1	
Obsessive compulsive disorder	1	0	
Illness duration	26.5 (13.9)	17.1 (10.7)	0.109
Age of onset	21.1 (6.3)	16.7 (7.1)	0.161
PANSS positive	13.9 (8.5)	11.1 (3.9)	0.389
PANSS negative	15.6 (10)	14.9 (5.7)	0.865
PANSS general	33.4 (16.2)	28.2 (5.5)	0.381
PANSS total	62.9 (33.0)	54.2 (12.1)	0.477
Chlorpromazine equivalent	576.0 (1082.5)	744.1 (667.2)	0.732
LSP total	120.1 (14.6)	118.8 (12.8)	0.812
NOSCA total	16.7 (1.9)	17.2 (1.5)	0.548
SNS total	17.6 (6.0)	18.1 (7.5)	0.861
CFQ total	35.2 (17.1)	38.2 (12.0)	0.640
Neuropsychological composite	−0.3 (0.8)	0.0 (0.5)	0.290

*Note*. CFQ = Cognitive Failure Questionnaire, CR = cognitive remediation, LSP = Life Skills Profile, NOS = not otherwise specified, NOSCA = Nurses Observation Scale of Cognitive Abilities, PANSS = Positive And Negative Syndrome Scale, PTSD = post-traumatic stress disorder, SNS = Self-report of Negative Symptoms, tDCS = transcranial direct current stimulation.

a The level of education is categorized using the Dutch Verhage scale (Verhage and Werff, 1964).

b Note that all participants had multiple comorbidities, and this is only an indication of the primary diagnosis.

### 3.2 Feasibility – retention and participation rates

Of the 24 participants, who were randomized and started the CR training, fifteen participants (62.5 %) completed at least 20 sessions and were considered treatment completers (see Table S7 for descriptive statistics comparing completers and non-completers). Participants attended on average 19.3 sessions (active tDCS group: M = 21.2 SD = 5.3, median = 22, range = 10–29; sham tDCS group: M *=* 16.6, SD = 7.7, median = 18, range = 1–28). More participants in the CR + active tDCS group completed the training (*n* = 10, 76.9 %) than in the CR + sham tDCS group (*n* = 5, 45.5 %).

### 3.3 Acceptability

#### 3.3.1 Theoretical framework of acceptability

Seventeen participants agreed to participate in the interview and their responses were coded into six of the seven TFA constructs (Table S2, Table S5). In the participants' responses, we did not identify any belief statements for the TFA construct “Ethicality” as participants struggled with answering the question about ethicality (“Do you think that this training is a good way to improve your quality of life?”).

The participants had a positive attitude towards the intervention, did not experience a big burden by the intervention, experienced low opportunity costs, and felt confident to perform the behaviors/skills learnt in the intervention on their own. Most participants (83.3 %) reported improvements on cognitive functioning, everyday functioning, or other skills, while they did not experience a burden by the tDCS, they were unsure whether it was an useful addition. The intervention coherence (i.e., the extent to which the participants understood the intervention and how it works) was low; most (83.3 %) participants struggled with explaining the rationale of the training. See the full results in Table 2 and Table S5.

**Table 2:** Acceptability belief statements and quotes of intervention evaluation interview.

Domain of Acceptability	Positive Belief Statement	Example quotes	Negative Belief Statement	Example quotes
**Affective Attitude** How an individual feels about the intervention, after taking part	I liked participating in the training. (*n* = 12) The training was interesting (because I learned new things). (*n* = 7) I liked that the training was personalized (*n* = 1) / was on the computer. (n = 1) **Would you recommend the training to others?** Yes (*n* = 14) - with brain stimulation (*n* = 6) - without brain stimulation (n = 1) - not sure about the brain stimulation (n = 6) There is no harm in trying. (*n* = 3)	*“Some things I could do well, and some not so well. I also didn't go to school, so I didn't learn these things. I really liked the little exercises though. I noticed that I felt a little bit more intelligent.”* *“It felt more like school than therapy. It made me feel like I had really learned something each time.”* *“I do know people who would benefit from improving their memory and concentration this way. “* *“I don't deal with other people that much, but I do recommend it. I would also recommend the brain stimulation, but I'm not quite sure.”*	I experienced a small discomfort by the tDCS, but I did not mind the brain stimulation. (*n* = 5) The tDCS was very burdensome. (*n* = 2) I am sad that the training ended. (*n* = 3) **Would you recommend the training to others?** No (*n* = 2)	*“[…], but at first it [the tDCS] was a little itchy”* *“I liked the training, but I hated the stickers (=rubber electrodes). Your head was all sticky after the training.”* *“I would not recommend the training to anyone else because I want to save them the trouble. I did enjoy the games, which I would also like to do with other friends.”* *“I would not recommend this training to anyone else because it had so little effect for me.”*
**Burden** The amount of effort that was required to participate in the intervention	The training intensity was good/it was intense, but not too intense. (n = 11) It was easy to motivate myself to go to the training. (*n* = 9) Some tasks were easy, others were difficult. Not too easy/too difficult. (*n* = 8)	*“Coming to training sessions twice a week was not difficult because I found it interesting. The motivation actually came naturally. I didn't find the intensity of the trainings to be too bad, but it shouldn't be longer because then you don't keep it up.”* *“I was able to motivate myself to go to the sessions. It also fit my schedule pretty well.”*	The training was too intense (too many sessions per week) (*n* = 4) Sometimes I found it difficult to motivate myself to go to the training. (*n* = 4) I found it hard to participate in the training because the tasks were too easy. (n = 1)	*“[…], but twice a week I found exhausting. Once you got there, it was good. In the end I am glad I did it.”* *“In the beginning I liked it, but later I found the tests a bit simple. Because it became too easy, I found it difficult to participate.”*
**Opportunity Costs** The benefits, profits, or values that were given up to engage in the intervention	I did not have to give up anything to participate in the training. (*n* = 16)		I had to cancel many appointments to participate in the training. (n = 1)	
**Intervention Coherence** The extent to which the participant understands the intervention and how it works	**Understand the goals of the training:** Improving everyday functioning/reaching personal goals (*n* = 7) **Able to explain how the training aims to improve everyday functioning** (n = 3) **Understanding of strategies:** Able to explain how strategies can help in everyday life (n = 1) Able to explain the purpose of using strategies (n = 3)		**Lack of/minimal understanding of the goals of the training:** Improving cognitive function/stimulating brain function (*n* = 9), see what is in your head (n = 1), reducing hallucinations (n = 1), I don't know (n = 1) **Lack of understanding how the training works:** focus on describing the tasks (n = 13) **Lack of understanding strategies:** Lists strategies without explanation (n = 6), help to perform better in training tasks (*n* = 5), I don't remember (much about) (n = 4)/ cannot explain the strategies (n = 1)	
**Perceived Effectiveness** The extent to which the intervention is perceived to have achieved its intended purpose	**Cognitive functioning** My thinking skills improved. (*n* = 11) I have more insight into (the strengths and weaknesses of) my thinking skills. (n = 5) **Everyday functioning** I observed improvements in my everyday life. (n = 13) The strategies I learnt in the training are useful in my everyday life/I use the strategies in my everyday life. (n = 6) **tDCS** I believe that the brain stimulation adds value to the training. (n = 4) **Other** I improved my computer skills. (n = 2) The training helped me to become less anxious (n = 1)/ helped me with my depression. (n = 1)	*“For example, when I go grocery shopping, I look at how long it takes me to do so. I never thought about this before. It is also easier in the store; I sometimes make a route through the store and I also make a list to remember things. I can also remember things better; for example, I can remember passwords better.”* *“I noticed that I take a little more rest when making decisions and I am more conscious of things. For example, I forget my room key less often. […] When I am in the supermarket, I check now what I am going to spend. Or I make a list in advance. I also divide tasks into chunks now, to make it more manageable.[…]. I don't know which brain stimulation group I was in, but I think it helped.”* *“What has especially helped me is taking a moment to think when I'm working on something. By paying a little more attention, I am able to remember things better. I've also become more accepting of my limitations, so I've also started keeping a calendar.”* *“I really got a lot out of the training, because I really found out that I can be anxious and unsure of my own abilities. I found out that it actually went pretty well!.”*	**Cognitive functioning** My thinking skills did not improve. (n = 2) My thinking skills improved less than I hoped for. (n = 1) I feel like improvements are disappearing now that I stopped with the training. (n = 2) **Everyday functioning** I did not observe improvements in my everyday life. (n = 3) I believe that I would have observed more improvements in my everyday life if I would have continued the training. (n = 2) **tDCS** I am not sure whether the brain stimulation added something to the training. (n = 11) **Other** The training did not help with my hallucinations. (n = 1)	*“The training has not helped with my thinking skills. At least I haven't noticed that yet.”* *“I didn't notice that much improvement after training. I still have to write everything down on notes and in a calendar”* *“My memory improved because I could remember numbers better. I think for a greater effect I needed to continue a little longer. Things in everyday life didn't necessarily get easier, many things I already do without effort.”* *“I notice that I can remember more things, and I noticed this during the tests. At other times during everyday life, I don't notice the difference yet. I don't know if the brain stimulation affected that, because I don't know if I had placebo.”* *“I don't know if the brain stimulation contributed to that, because I don't know anything about that. I think most of the improvement came from the training.”*
**Self-Efficacy** The participant's confidence that they can perform the behavior(s) required to participate in the intervention	I believe that I can perform the training tasks independently. (*n* = 12) I gained more self-confidence in performing the tasks during the training (n = 8)	*“At first, I was nervous to do the tasks, but later I thought I could handle it. So my self-confidence increased the more trainings I did.”* *“I had confidence in it [performing the tasks]. After the training, I feel able to do the tasks independently as well.”*	I believe that I need help to perform the training tasks. (n = 3)	*“I felt confident that I would be able to do the computer tasks. Without the therapist, I don't think I could do the training. It became easier because she was there.”*

*Note.* The belief statements were coded from the intervention evaluation interviews of 18 participants. The n's within the acceptability domains do not add up to 18, as some participants made multiple belief statements within one domain. Participants' responses in the intervention evaluation interview were coded into six of the seven TFA constructs. In the participants' responses, we did not identify any belief statements for the TFA construct “Ethicality” as participants struggled with answering the question about ethicality (“Do you think that this training is a good way to improve your quality of life?”).

#### 3.3.2 Additional results intervention evaluation interview

Three out of 17 participants who completed the interview could state the specific goal discussed with the therapist. Eight participants mentioned goals that were related to cognitive skills, symptoms, or other factors (e.g., computer skills, increasing self-confidence, being less impulsive) but did not formulate these in a specific, measurable manner. Three participants stated that they did not have goals during the training.

#### 3.3.3 Side effects

Most (68.4 %) participants did not experience any or experienced mild side effects from the tDCS (Table 3). The frequency and severity of the sensations experienced by participants were similar in the sham tDCS and active tDCS group. In relation to the cognitive remediation, participants did not report any side effects.

**Table 3:** Adverse effects related to tDCS.

Adverse effect	CR + sham tDCS (*n* = 7)		CR + active tDCS (*n* = 12)	
	*n* (%)	Severity median (range)	*n* (%)	Severity median (range)
Itch	5 (71.4 %)	1 (1–2)	5 (41.7 %)	1 (1–3)
Pain	3 (42.9 %)	2 (1–2)	3 (25.0 %)	1 (1–2)
Burn	4 (57.1 %)	1 (1–3)	4 (33.3 %)	1 (1–3)
Warmth/heat	3 (42.9 %)	1 (1–2)	3 (25.0 %)	1 (1–2)
Metal taste	1 (14.3 %)	1	0	–
Fatigue	0	–	2 (16.7 %)	1

*Note.* Adverse effects reported from participants in the last session reflecting on the whole training. The severity was ranked from 1 to 3 (1 = mild, 2 = moderate, 3 = severe). Five participants did not report the adverse effects, as three participants were not attending the last session(s) due to admission to a psychiatric hospital/severity of symptoms and two participants did not receive tDCS.

### 3.4 Preliminary effectiveness

The results of all tested models, means and standard deviations per outcome measure, time point and treatment condition are available in the online supplements (https://osf.io/cj49e/).

#### 3.4.1 Main effect CR

In Table 4, the post-intervention effects and 6-month follow-up effects are presented from the model with the best model fit (based on the Akaike Information Criteria, AIC). The neurocognitive composite improved significantly post-treatment (*t*(74) = 4.98, *p <* .001) and sustained improvements were observed at 6-month follow-up (*t*(74) = 4.28, *p <* .001). The neurocognitive composite did not improve from baseline to post-waiting period (effect was excluded in final model). Exploratory analyses for each neurocognitive test are available in the online supplements (https://osf.io/cj49e/) and showed improvements in the domains verbal learning, visual learning, working memory, and reasoning and problem solving, not for attention/vigilance and speed of processing. Self-perceived negative symptoms (SNS) did not improve significantly post-treatment but showed significant improvements at 6-month follow-up (*t*(71) = −2.57, *p* = .012). Interestingly, for the observer-rated everyday functioning (LSP), we found a significant improvement of the LSP after the waiting period (*t*(71) = −2.37, *p* = .020) but not post-treatment or at the 6-month follow-up. Neither the observer-rated cognitive functioning (NOSCA) nor the self-reported cognitive functioning (CFQ) showed significant improvements at post-treatment or 6-month follow-up.

**Table 4:** Fixed and random effects of model comparing cognitive remediation vs. treatment as usual.

Parameter	LSP	NOSCA	Global Cognition	CFQ	SNS
Fixed effects - Beta (SE)					
Intercept	124.45 (2.34)⁎⁎	17.10 (0.27)⁎⁎	−0.13 (0.13)	35.15 (3.00)⁎⁎	18.69 (1.29)⁎⁎
T1 vs. T0 (TAU)^a^, ^b^	−4.92 (2.14)⁎	…	…	…	…
T1 vs. T2 (CR)	2.60 (2.29)	0.18 (0.37)	0.26 (0.05)⁎⁎	−1.13 (2.20)	−1.51 (1.27)
T1 vs. T3	1.76 (2.57)	0.01 (0.42)	0.31 (0.07)⁎⁎	−2.44 (2.57)	−3.83 (1.45)⁎
Random effects - variance (SD)					
Level 3 – slopes^b^	…	…	0.01 (0.08)	…	…
Level 2 – intercept	73.44 (8.57)	0.80 (0.90)	0.39 (0.63)	184.88 (13.59)	26.99 (5.20)
Level 1 – residual	50.32 (7.09)	1.65 (1.28)	0.03 (0.17)	57.42 (7.58)	20.28 (4.50)
Model fit					
Log-Likelihood	−280.49	−136.06	−22.64	−290.39	−241.26
AIC	559.49	283.18	69.64	579.69	485.44

Note. CFQ = Cognitive Failure Questionnaire, CR = cognitive remediation, LSP = Life Skills Profile, NOSCA = Nurses Observation Scale for Cognitive Abilities, SNS = Self-Report of Negative Symptoms, TAU = treatment as usual, tDCS = transcranial direct current stimulation; etc. SD = standard deviation, SE = standard error. For the LSP, NOSCA and global cognition (standardized), a higher score represents a more favorable outcome. For the CFQ and SNS, a lower score represents a more favorable outcome.

a Note that the betas were calculated compared to the reference level T1. Hence, a negative value for T1 vs. T0 indicates an increase of the outcome variable from T0 to T1, while a positive value for T1 vs. T2 and T1 vs. T2 indicates an increase of the outcome variable. For example, the model predicted an increase of 4.92 points from T0 to T1, and an increase of 2.6 points from T1 to T2.

b … = effect appeared not to be significant (fixed effect) or did not increase the model fit (random fit) and was therefore removed from the model. For each outcome measure, the model with the best model fit according to the AIC is presented.

⁎ *P* < .05.

⁎⁎ *P* < .001.

#### 3.4.2 CR + sham tDCS vs. CR + active tDCS

Table 5 presents the results of the model including tDCS as a predictor. None of the outcome measures revealed significant group differences between the CR + sham tDCS group and the CR + active tDCS group.

**Table 5:** Fixed and random effects of model including tDCS condition as predictor.

Parameter	LSP	NOSCA	Global Cognition	CFQ	SNS
Fixed effects - Beta (SE)					
Intercept	125.69 (3.10)⁎⁎	17.12 (0.38)⁎⁎	−0.27 (0.19)	35.92 (4.26)⁎⁎	20.55 (1.95)⁎⁎
T1 vs. T0 (TAU)^a^, ^b^	−4.97 (1.96)⁎	…	…	…	…
T1 vs. T2 (CR)	2.63 (2.13)	0.18 (0.38)	0.26 (0.05)⁎⁎	−1.09 (2.21)	−1.60 (1.24)
T1 vs. T3	1.06 (2.77)	−0.02 (0.44)	0.37 (0.07)⁎⁎	−2.40 (2.67)	−3.85 (1.61)⁎
tDCS	−2.22 (3.85)	−0.03 (0.49)	0.27 (0.25)	−1.20 (5.77)	−2.78 (2.40)
Random effects - variance (SD)					
Level 3 – slopes	9.85 (3.14)	0.05 (0.22)	0.01 (0.08)	1.72 (1.31)	3.36 (1.83)
Level 2 – intercept	99.65 (9.98)	0.78 (0.89)	0.37 (0.61)	166.15 (12.89)	44.66 (6.68)
Level 1 – residual	38.20 (6.18)	1.58 (1.26)	0.03 (0.17)	55.23 (7.43)	15.37 (3.92)
Model fit					
Log-Likelihood	−279.29	−135.99	−22.13	−290.09	−239.38
AIC	558.76	288.54	71.09	579.66	484.10

Note. CFQ = Cognitive Failure Questionnaire, CR = cognitive remediation, LSP = Life Skills Profile, NOSCA = Nurses Observation Scale for Cognitive Abilities, SNS = Self-Report of Negative Symptoms, TAU = treatment as usual, tDCS = transcranial direct current stimulation; etc. SD = standard deviation, SE = standard error, … = effect appeared not to be significant (fixed effect) or did not increase the model fit (random fit) and was therefore removed from the model. For each outcome measure, the model with the best model fit according to the AIC is presented.

a Note that the betas were calculated compared to the reference level T1. Hence, a negative value for T1 vs. T0 indicates an increase of the outcome variable from T0 to T1, while a positive value for T1 vs. T2 and T1 vs. T2 indicates an increase of the outcome variable. For example, the model predicted an increase of 5.07 points from T0 to T1, and an increase of 2.6 points from T1 to T2.

b … = effect appeared not to be significant (fixed effect) or did not increase the model fit (random fit) and was therefore removed from the model. For each outcome measure, the model with the best model fit according to the AIC is presented.

⁎ *P* < .05.

⁎⁎ *P* < .001.

Additional results regarding blinding and the impact of COVID-19 are available in the supplements.

## 4 Discussion

This randomized controlled pilot trial showed that combining CR and tDCS was feasible and acceptable for 24 participants with SMI. A total of 62.5 % completed at least 20 CR sessions, meeting the 60 % retention rate. Participants were positive about the training, reported subjective cognitive and everyday life improvements, and would recommend it to others. Small, statistically significant cognitive improvements were observed after 4 months of CR compared to stable performance during a 4-month waiting period, and these improvements persisted 6 months post-treatment. Additionally, negative symptoms improved 6 months post-treatment. However, observer-rated everyday functioning and cognitive functioning did not significantly improve. Participants experienced few, mostly mild side effects from tDCS. No differences in outcomes were found between those who received CR with active tDCS and those with sham tDCS.

This study builds upon previous findings regarding the acceptability of CR for inpatients with psychosis, extending its application to a broader range of individuals with severe mental illnesses in long-term psychiatric care, encompassing extended hospital stays and sheltered housing. Most service users liked participating in the training (70.5 %), did not find it burdensome (76.4 %), and recognized personal benefits (88.2 %) without experiencing any side effects. Among interviewed participants, 70.6 % reported tangible improvements in everyday life, such as reading books, navigating supermarkets, and pursuing higher education.

However, these improvements were not reflected in the outcome measures we used. Prior research suggests that CR combined with other psychosocial interventions shows more impact on complex everyday functioning, such as social or vocational functioning, rather than community functioning (Duin et al., 2019). Our chosen outcome measure, the LSP, might not have been sensitive enough to detect these changes due to its broad scope. Future studies should consider alternative outcome measures (for an overview of potential outcome measures, see Searle et al., 2022) or personal goal attainment assessments to better capture meaningful improvements in everyday functioning.

Throughout the interviews an array of themes emerged that hold relevance for the integration of CR in future research and clinical applications tailored to individuals within long-term psychiatric clinical care. Firstly, participants' views on session frequency varied; some found two sessions a week too intense, whereas others were comfortable with this, and others even wished for extended training. Therapists observed that certain participants needed more time to gain confidence in task execution before they were able to discuss application of skills to everyday life, suggesting a potential benefit from longer training periods.

Secondly, some participants did not understand the intervention's purpose or how it applied to everyday life, indicating a need for therapists to clarify the treatment's rationale and relevance. Additionally, most participants did not have specific goals for CR training and struggled with collaboratively identifying personally relevant goals with the therapist, which is a crucial aspect of the program. This challenge, noted in previous studies in the same population (see e.g., Stiekema et al., 2020), may be more pronounced in inpatient settings where individuals find it difficult to set or work on personal goals due to the limited range of pleasurable activities available. To address this in future studies, training CR therapists in goal-setting for inpatients or preceding CR with goal-formulation interventions may be beneficial.

### 4.1 Effects on cognition, negative symptoms, and everyday functioning

Neuropsychological test scores improved following CR, with small improvements persisting 6 months post-treatment and none seen after the waiting period, ruling out practice effects. Specifically, improvements were noted in verbal learning, visual learning, working memory, and reasoning and problem-solving, but not in attention/vigilance and speed of processing. These findings align with previous meta-analyses on CR's effectiveness for cognitive outcomes in schizophrenia (Vita et al., 2021; Cella et al., 2020) and extend them to individuals with severe mental illness in long-term care.

However, cognitive improvements did not transfer to observer-rated everyday functioning, consistent with some studies (e.g., Dark et al., 2022) but contrasting others focused on CIRCuiTS (e.g., Wykes et al., 2023). No significant improvements were seen in self-report and observer-rated questionnaires on everyday cognitive functioning. This might be due to the need for longer training, goal-setting struggles, therapist difficulties in discussing skill transfer, or inadequacies in the chosen outcome measures. For instance, the self-report questionnaire focused on attention and memory, while many participants noted subjective improvements in planning, suggesting the questionnaire missed some improvement areas.

This study showed improved negative symptoms not immediately after the treatment but at the 6-month follow-up. The post-treatment improvements may have been too small to detect in this small study. A previous meta-analysis found a small effect size of 0.3 for improved negative symptoms post-CR, requiring 120 individuals per group for statistical significance (Cella et al., 2017). The continuous improvement of negative symptoms could be due to increased behavioral activation following CR. Although we did not measure this, multiple participants reported engaging in more activities or spending more hours in education or work after the intervention.

We found that participants observed everyday functioning improved significantly during the waiting period (before starting the CR training). A possible explanation could be that service users started to participate in the study once they developed readiness for rehabilitation (Cohen et al., 1997). Anecdotal evidence from recruitment suggested that service users were often referred by their case manager once their treatment plan was changed to include more activities in general. While not formally tracked, this increase in activity could explain the observed improvements. Furthermore, participation in the study itself and the expectation of benefit, or insights from baseline assessments, may have enhanced functioning. Yet, due to the study's limited size, these findings could be coincidental and need more research.

### 4.2 Current evidence for combining cognitive remediation and tDCS

Most participants reported only minor side effects, which did not affect their performance during CR. Overall, the tDCS intervention was well-received: six out of seventeen interviewed participants recommended the combined approach, while eleven were uncertain about tDCS' added value. Therapists shared mixed opinions, noting that while tDCS preparation required extra time and was somewhat impractical, they would not mind the effort if tDCS proved effective. Both participants and therapists found the small additional burden acceptable if tDCS delivered significant benefits. This finding exemplifies the importance of using clinical relevant outcome measures that participants and therapists find meaningful in future studies to increase the likelihood of implementation into clinical practice. This study did not find significant benefits of adding tDCS to CR, likely due to the small sample size and expected improvements from CR alone.

Interestingly, fewer participants in the sham tDCS group completed the intervention and attended five fewer sessions on average. This may be due to differences between the groups: the sham group was older (sham: 45.4 ± 16.1 vs. active: 33.1 ± 12.6) and had a longer illness duration (sham: 26.5 ± 13.9 vs. active: 17.1 ± 10.7). Non-completers were also older (non-completers: 53.3 ± 14.0 vs. completers: 30 ± 7.4) and had a longer illness duration (non-completers: 30.8 ± 12.8 vs. completers: 15.8 ± 9.6) across both groups. These discrepancies highlight the need for larger trials to evaluate the effectiveness of combining tDCS and CR in clinical practice.

### 4.3 Strengths and limitations of the current study

This trial is one of the first to evaluate the combination of CR and tDCS in people with SMI in long-term psychiatric clinical care. Particularly, the focus on improving everyday functioning using CR with the four core elements with a strong evidence base is a strength in this study. Another strength of the trial is the combination of quantitative and qualitative measures.

Some limitations should be considered. Firstly, this study did not include a condition controlling for the contact between the participant and the therapist. The waiting period (treatment as usual) allowed us to evaluate the outcomes in a within-subject design, which had our priority in this pilot trial given the diversity of the sample in this transdiagnostic population. Secondly, the trial was conducted during the COVID-19 pandemic. Most participants experienced lockdowns and other COVID-related restrictions during the trial, which might have influenced the results regarding everyday functioning. Thirdly, the small sample size limited the statistical power to detect differences between the active and the sham tDCS condition. Fourthly, one therapist (AP) correctly identified five participants in the active tDCS group due to skin redness, a known limitation of blinding with sham tDCS (Palm et al., 2013). However, AP did not conduct outcome assessments, so assessment blinding was maintained. Lastly, we rated everyday functioning using only one observer-rated questionnaire. For some participants, the same person completed all assessments, but for most, different people did. This variation in observers could have influenced the outcomes. Due to the high turnover of mental health staff, future studies should use additional measures beyond observer-rated questionnaires to obtain a fuller picture.

### 4.4 Conclusion

This pragmatic pilot trial demonstrated the acceptability and feasibility of combining CR and tDCS for people with severe mental illness in long-term psychiatric clinical care. CR appeared to yield self-reported benefits to participants in addition to small cognitive improvements on the test battery in the short- and longer-term and improved negative symptoms at 6-month follow up. However, observed everyday functioning and cognition, as well as subjective cognitive complaints, did not change. While adding tDCS was not a significant burden, future studies are needed to determine if tDCS can enhance CR treatment outcomes.

## CRediT authorship contribution statement

**Anika Poppe:** Writing – original draft, Visualization, Project administration, Investigation, Formal analysis. **Leonie Bais:** Writing – review & editing, Supervision, Funding acquisition, Conceptualization. **Daniëlle van Duin:** Writing – review & editing, Supervision, Funding acquisition, Conceptualization. **Branislava Ćurčić-Blake:** Writing – review & editing, Supervision, Funding acquisition, Conceptualization. **Gerdina Hendrika Maria Pijnenborg:** Writing – review & editing, Supervision, Funding acquisition, Conceptualization. **Lisette van der Meer:** Writing – review & editing, Supervision, Funding acquisition, Conceptualization.

## Declaration of Generative AI and AI-assisted technologies in the writing process

During the preparation of this work the authors used ChatGPT to improve readability and language. After using this tool, the authors reviewed and edited the content as needed and take full responsibility for the content of the publication.

## Funding

This study was supported by 10.13039/501100007502Stichting tot Steun VCVGZ (grant number 264). The funder had no role in the study design; in the collection, analysis, and interpretation of the data; in writing of the report, or in the decision to submit the article for publication.

## Declaration of competing interest

None.

## Appendix A Supplementary data

Supplementary material Image 1
