# Supplementary material for: Feasibility and acceptability of combining cognitive remediation and tDCS in long-term psychiatric clinical care
Source: Schizophr Res Cogn. 2025 Jun 10;42:100358. doi: 10.1016/j.scog.2025.100358 (PMC12182772; doi:10.1016/j.scog.2025.100358)
Supplement: Supplementary file 1 — Supplementary material [file mmc1.docx]

**Supplementary material**

**Feasibility and Acceptability of Combining Cognitive Remediation and tDCS in Long-Term Psychiatric Clinical Care**

Anika Poppe (corresponding author, a.poppe@rug.nl)^1,2^, Leonie Bais^3^, Daniëlle van Duin^4^, Branislava Ćurčić-Blake^5^, Gerdina Hendrika Maria Pijnenborg^1,6^, Lisette van der Meer^1,2^

Affiliations:
^1^Department of Clinical and Developmental Neuropsychology, University of Groningen, Grote Kruisstraat 2\1, 9712 TS Groningen, The Netherlands. ^2^Department of Rehabilitation, Lentis Psychiatric Institute, Lagerhout E35, 9741 KE Zuidlaren, The Netherlands. ^3^Formerly at Department of Rehabilitation, Lentis Psychiatric Institute, Lagerhout E35, 9741 KE, Zuidlaren, The Netherlands. ^4^Phrenos Center of Expertise, Utrecht, The Netherlands. ^5^Department of BSCS Neuroscience, University of Groningen, University Medical Center Groningen, Groningen, The Netherlands. ^6^Department of Psychotic Disorders, GGZ Drenthe, Assen, The Netherlands.

Influence of the researchers – reflexivity 4

Data availability statement 4

Recruitment 4

Randomization and Blinding 4

Baseline measures 5

Current Flow Modelling 6

Additional results 8

Table S2. Topics and questions covered in the semi-structured interview 10

Table S3. Description outcome measures 13

Table S4. Participants’ motivations to engage in the study 16

Table S5. Belief statements intervention evaluation interview 17

Table S6. Standardized effect sizes [95% CI] for the main analyses 20

Table S6. Standardized effect sizes [95% CI] for the main analyses including tDCS 21

Table S7. Baseline characteristics completer vs. non-completer 22

Table S8. Baseline characteristics comparison between participants with prolonged waiting periods due to COVID-19 and other participants 24

Table S9. Comparison of changes during the waiting period between participants with prolonged waiting periods due to COVID-19 and other participants 25

References 27

## Influence of the researchers – reflexivity

When writing this manuscript, we recognize the significance of our personal engagement throughout this study’s process, which inevitably shaped the nuanced interpretation of the (qualitative) outcomes. Notably, AP undertook the role of CR therapist for most participants and took the lead in composing the initial draft of the manuscript. LB and LvM conducted the interviews, and GHMP and DvD were the supervisors of the CR therapists. Through our multifaceted involvement, we aim to enrich the authenticity and depth of our reported findings.

## Data availability statement

The datasets generated and/or analyzed during the current study are not publicly available. Under the General Data Protection Regulation (GDPR), our data is considered pseudonymized rather than anonymized and is, therefore, still regarded as personal data. Given that participants have not given informed consent to have their personal data publicly shared, we are legally and ethically not allowed to publicly post our dataset. The dataset is available from the corresponding author on reasonable request.

## Recruitment

Participants were recruited from a long-term psychiatric hospital or sheltered living facility of Lentis Psychiatric Institute (Northern Netherlands). We organized information meetings for service users in the treatment facilities and informed treating nurses, psychologists, and psychiatrists about the study aims and eligibility criteria. Eligible, interested service users were further informed about the project by one of the researchers and received written information. After a one-week consideration period, participants signed the informed consent. During the 16-week waiting period, the CR therapist reached out to the participants once a month to keep the participants engaged in the study. This procedure was established to minimize drop-outs during the waiting period.

## Randomization and Blinding

***Sequence Generation*** Participants were randomly assigned to CR + sham tDCS (group 1) or CR + active tDCS (group 2) with a 1:1 allocation as per computer-generated randomization plan using permuted blocks of random sizes (2, 4, 6). The block sizes were not disclosed to ensure concealment.

***Concealment Mechanism*** A randomization sequence was generated by a randomization plan generator (http://randomization.com). The tDCS device is pre-programmed with codes that are linked to either real or sham stimulation. Twenty-six codes, which are printed in the manual, were selected randomly (13 for sham stimulation, 13 for real stimulation) and linked to the randomization sequence. The manual page with the codes and the randomization sequence was kept in two separate sealed envelopes which will remain closed until the completion of the data collection. Only the sequence of the codes were accessible during the trial, which cannot be linked to real or sham stimulation without the randomization sequence or the manual page. When participants completed the second baseline measurement (T1), the first available code in the sequence was coupled to the participant number.

***Implementation*** An independent researcher generated the allocation sequence, selected the tDCS codes, linked them to the randomization sequence and sealed the envelopes. This researcher was not involved in any other part of the study and did not work in the research group by the time the trial commenced. The investigators enrolled participants, linked the tDCS code to the participant number following the second baseline measurement (T1), and communicated the code to the CIRCuiTS therapist. The randomization sequence and the block sizes were not disclosed until all participants completed the follow-up measurement (T3). Thus, randomization was conducted without any influence of the investigators, raters or therapists.

***Who will be Blinded*** Using the allocation procedure described above the trial was triple-blind to the tDCS experimental condition; the investigators, the CIRCuiTS therapist and the participant stayed blind to the tDCS treatment condition. The CIRCuiTS therapist and participant were asked to indicate whether the participant received either active or sham stimulation in session 10 of the intervention and at T2. The participants were informed about the type of treatment they have received after completion of the trial. As all participants received CIRCuiTS, the investigators, CIRCuiTS therapist and participants were not blind to the CIRCuiTS experimental condition. Research assistants (at least BA level psychologists) were trained to administer the assessments. They will be blind to the experimental condition (both tDCS and CIRCuiTS) of the participants, as they will not receive any information regarding the aims and design of the trial, nor of the intervention.

## Baseline measures

We collected demographical information (age, sex, level of education, primary diagnosis, illness duration, age of onset, chlorpromazine equivalent), Positive and Negative Syndrome Scale (PANSS) (Kay et al., 1987) scores from the yearly Routine Outcome Monitoring screening (with participants’ consent). We confirmed that the scores from the last PANSS were representative of the individuals’ current symptoms with their treating clinician. If the PANSS scores were not representative or available, a trained researcher or research assistant administered the PANSS. We additionally assessed participants’ motivations to participate and their expectations regarding the intervention effectiveness by means of a brief questionnaire containing open questions (i.e., Do you believe that the training can improve your own or others cognitive and/or everyday functioning? Why did you decide to participate in the study?).

## Current Flow Modelling

The electrode locations were determined with computational modelling using the SimNIBS software (Saturnino et al., 2019). With computational modeling, the current flow resulting from a given electrode montage can be calculated using realistic model heads or structural MRI scans. We used computational modeling to compare different electrode montages that are commonly used and would most likely target the left dorsolateral prefrontal cortex (DLPFC). We chose the left DLPFC as it is an important hub of the fronto-parietal networks, which are important for the execution of cognitive tasks (Cole et al., 2013) and are often affected in people with a diagnosis in the schizophrenia spectrum (Sheffield et al., 2015). For the models, we used MRI scans from five participants from an earlier study that was conducted in the same target population. We chose these participants’ MRIs based on their body mass index (BMI); one being underweight, two average weight, and two being overweight. The BMI was chosen as a proxy of subcutaneous fat level; which is one of the physiological factors influencing the current flow (Li, 2015). Table S1 shows the mean electric field estimated in the left DLPFC. The strongest mean electric field was estimated when the anode was placed on C3 and the cathode was placed on Fp1. Figure S1 presents the resulting electric field for one of the subjects.

| **Table S1.** Mean electric fields in the left DLPFC (-38, 44, 26) in V/m | | | | | |  |
| --- | --- | --- | --- | --- | --- | --- |
| Subjects | C3-Fp1 | C3-Fp2 | C5-Fp1 | C5-Fp2 | F3-F4 | F3-Fp2 |
| S1 | 0.4306 | 0.3780 | 0.3871 | 0.3700 | 0.2261 | 0.2355 |
| S2 | 0.4093 | 0.3528 | 0.3653 | 0.3392 | 0.1695 | 0.1769 |
| S3 | 0.3866 | 0.3498 | 0.3404 | 0.3397 | 0.2129 | 0.2210 |
| S4 | 0.3444 | 0.3125 | 0.3036 | 0.3058 | 0.2098 | 0.2051 |
| S5 | 0.3434 | 0.3152 | 0.3054 | 0.3050 | 0.1880 | 0.1999 |


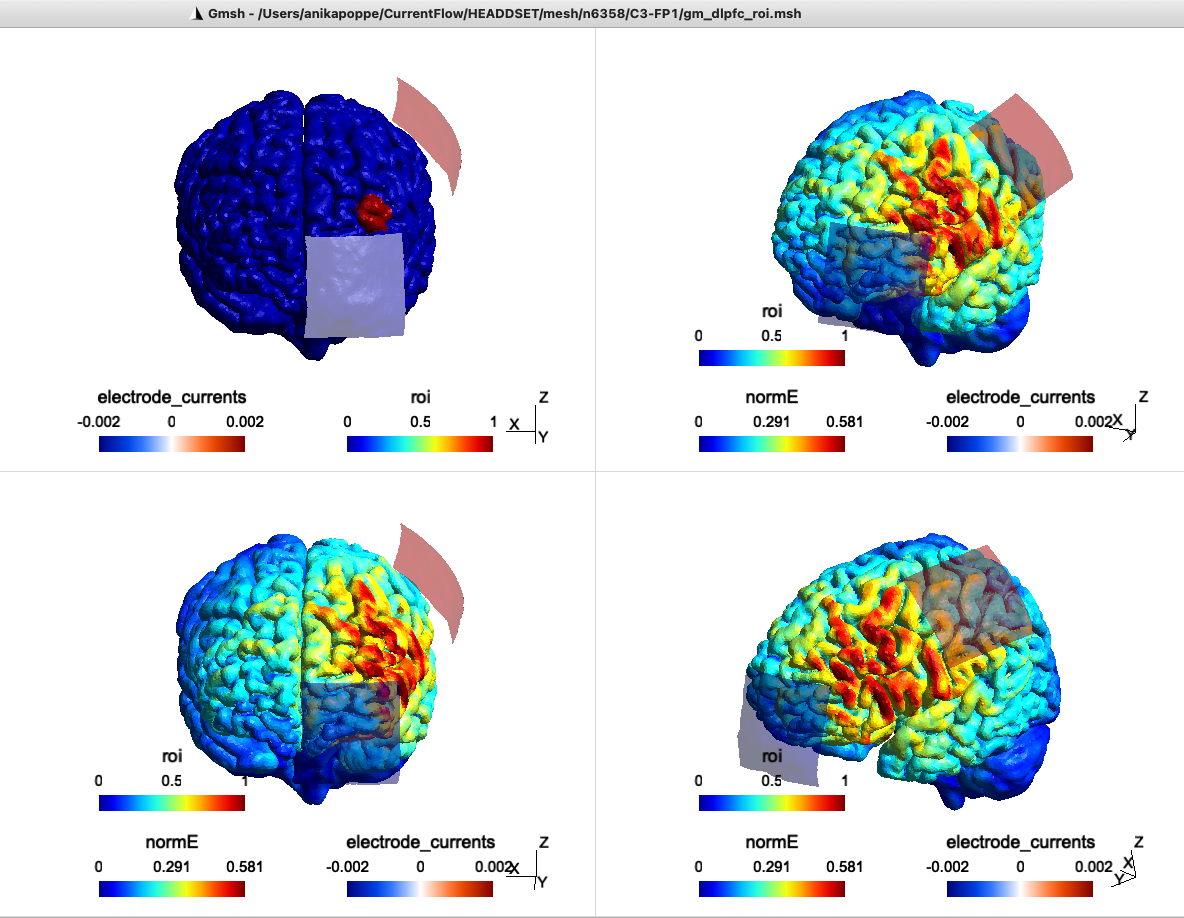


Figure 1. Electric field C3-Fp1

## Additional results

### Blinding

At the end of the treatment period, the participants expected to be in the active tDCS group (sham tDCS: n = 4; active tDCS: n = 6) or had no idea in which group they were (sham tDCS: n = 3; active tDCS: n = 6). The therapists indicated to have no idea about the treatment condition of 15 participants (sham tDCS: n = 7; active tDCS: n = 8) and guessed correctly that the remaining 5 participants received active tDCS.

### Impact of COVID-19

This trial was conducted during the COVID-19 pandemic, necessitating adjustments to align with national COVID-19 regulations. All assessments and training sessions were conducted in person. Due to national lockdowns, the training for seven participants was delayed beyond the 16-week waiting period, resulting in a total waiting period for these participants between five to eight months. During the waiting period, there was no difference in outcomes between the seven participants and the rest, except in the CFQ, with the seven showing a decline in subjective cognitive functioning, unlike the remaining seventeen who showed improvement. For further details, see Tables S8 and S9, which compare descriptive statistics and changes during the waiting period. Removing participants with longer waits did not impact results except for a significant improvement in subjective cognitive functioning. Detailed findings are in the online supplementary materials (osf.io).

## Table S2. Topics and questions covered in the semi-structured interview

| Theoretical Framework of acceptability (TFA) | Definition | Questions |
| --- | --- | --- |
| Ethicality | The extent to which the intervention has good fit with an individual’s value system | Do you think that this training is a good way to improve your quality of life? |
| Affective  Attitude | Experienced Affective Attitude: How an individual feels about the intervention, after taking part | How did you like participating in this training? Why?  Would you recommend this training to someone else?   - With or without brain stimulation? |
| Burden | Experienced burden: the amount of effort that was required to participate in the intervention | In your opinion how easy or difficult was it to participate in this intervention?  Did you have trouble motivating yourself to participate in the training sessions? If yes, for what reasons?  How intense did you find the training (referring to time investment)? |
| Opportunity  Costs | Experienced opportunity cost: the benefits, profits or values that were given up to engage in the intervention | Did you have to give up anything (or were you not able to do something) to be able to participate in the training? |
| Perceived  effectiveness | Experienced effectiveness: the extent to which the intervention is perceived to have achieved its intended purpose | In your opinion to what extend do you think the training in general was able to help you improve your cognitive functioning (e.g., name examples of cognitive functions that could have been improved)?  In your opinion to what extend do you think the training in general was able to help you to do things easier in your everyday life?  Do you think that the brain stimulation influenced these effects?  Do you think that this intervention could work in a similar way for someone else (i.e., improve cognition or not improve cognition; help or not help in everyday life)? |
| Self-efficacy | The participant's confidence that they can perform the behaviour(s) required to participate in the intervention | Do you think you will be able to perform some of the skills you learned in the training on your own now that the training ended?  Did you feel confident to perform the activities that you were asked to in the training? |
| Intervention Coherence | The extent to which the participant understands the intervention and how it works | Can you tell me a bit about the intervention itself?  What did you do during the sessions?  How does the training work?  You used strategies during the training, can you tell me more about the strategies?  What was the goal of the training? |

| Other topics | Definition / Explanation | Questions |
| --- | --- | --- |
| Relationship with Therapist |  | Which aspects did you appreciate about the therapist?  Was there something that you did not like about your therapist?  Do you think the training could have been more/less helpful if you would have had another therapist? |
| Goals |  | Did you work on goals during the training?  Did you set these goals on your own, did your therapist set the goals, or did you set them together?  What kind of goal(s) were you working on?  Were these goals important to you? |

## Table S3. Description outcome measures

| **Outcome domain** | **Measure** | **Description** | **Scoring** |
| --- | --- | --- | --- |
| Observer-rated everyday functioning | **Life Skills Profile**  (Rosen et al., 1989) | Questionnaire consisting of 39 questions that are scored on a 4-point scale. The questionnaire is developed from a positive mental health philosophy, by emphasizing “life skills” rather than “lack of life skills” and measures a range of aspects related to successful community or hospital living: (1) self-care, (2) non-turbulence, (3) social skills, (4) communication, and (5) responsibility. The was completed by the participant’s case manager | Total score (0-156) |
| Observer-rated cognitive functioning | **Nurses Observation Scale for Cognitive Abilities (NOSCA)**  (Persoon et al., 2012) | Behavioral rating scale to examine cognitive abilities. The scale includes 39 items that are scored on a 4-point scale, and it comprises eight subscales: attention, perception, memory, orientation, thoughts, language, and praxis. The NOSCA was completed by the participant’s case manager. | Total score (0-121) |
| Self-reported cognitive functioning | **Cognitive Failure Questionnaire (CFQ)**  (Broadbent et al., 1982; Ponds et al., 2006) | Self-report questionnaire that measures subjective cognitive functioning. The scale includes 25 questions that represent the cognitive subdomains of attention and memory. | Total score (0-100)* |
| Self-reported negative symptoms | **Self-report of Negative Symptoms (SNS)** (Dollfus et al., 2019) | Self-report questionnaire contains 20 items covering the five domains of negative symptoms (social withdrawal, diminished emotional range, avolition, anhedonia, and alogia) and were scored on a three-point Likert scale, based on the feelings during the previous week. | Total score (0-40)* |
| **Cognitive functioning assessed with neuropsychological test battery** | | | |
| Processing speed | **Controlled Oral Word Association Test (COWAT)**  (Schmand et al., 2008) | Participants name as many words as possible within a time span of 60s beginning with a given letter. The test is repeated for three different letters. | Total correct words named in three trials |
| Attention | **WAIS-IV Digit Span Forward** (Wechsler, 2008) | The test leader reads out a sequenc of digits and the participant is asked to repeat the numbers in the same order. The digit span increases every second trial. | Total number of correct trials (0-16) |
| Working memory | **WAIS-IV Digit Span Backward** (Wechsler, 2008) | Same as WAIS-IV Digit Span Forward, but the participant is asked to repeat the numbers in reversed order | Total number of correct trials (0-14) |
| Visual learning/memory | **Rey Complex Figure Text** (Osterrieth, 1944; Rey, 1941) | The participant is asked to copy a complex figure (copy trial). Thirty minutes later, the participants is asked to draw the complex figure from memory (recall trial). The figure is scored based on which elements were correctly drawn and correctly placed. | Total score from recall trial (0-36) |
| Verbal learning/memory | **15-word learning task (15-WT)**  (Saan & Deelman, 1986) | The test leader reads 15 words out loud and the participant is asked to recall as many words as possible. This procedure is repeated five times (learning trials). After continuing with the test battery for 15 min, the participant will be asked to name as many words as they can remember (recall trial). | Total score learning trials (0-65)  Total score recall trial (0-15) |
| Reasoning and problem solving | **Modified Card Sorting Test (MCST)** (Nelson, 1976; Stroop, 1935) | The test leader presents four stimulus cards with different shapes and colors. Participants receive a pile of 48 cards and are asked to lay down the cards one by one on the four presented cards, following a rule that the participant has to discover. The MSCT is a simplified version of the Wisconsin Card Sorting Test. | Categories completed (0-6)  Number of perseverative errors* |
| Reasoning and problem solving  Processing Speed | **Stroop Color and Word Test (SCWT)** (Nelson, 1976; Stroop, 1935) | Participants are asked to read out three cards as fast as possible. The first card contains words of colors and participants have to read them out (Word trial). The second card contains squares in different colors, and the participant has to name the color (Color trial). The last card contains words of colors (e.g., the word ‘red’) which are printed in different colors (e.g., the word ‘red’ printed in green ink). The participant has to name which color the ink of each word has (Color-Word trial). | Reaction time in Word trial (processing speed)*  Interference score *  = Color/Word RT – ((Color RT + Word RT)/2)  (Reasoning and problem solving) |

Note. RT = reaction time
*lower score represents a more favorable outcome (if scores are not marked with an asterisk, higher scores represent more favorable outcomes)

## Table S4. Participants’ motivations to engage in the study

| Motivation | n |
| --- | --- |
| Curiosity/like to do new things | 6 |
| Interesting | 5 |
| Support research | 5 |
| Train cognition | 4 |
| Money | 4 |
| Hope it helps | 3 |
| Learn something | 1 |
| Get better and less deterioration of the brain | 1 |
| See whether stimulation works | 1 |

## Table S5. Belief statements intervention evaluation interview

| **Statement** | **Total** | **active tDCS** | **sham tDCS** |
| --- | --- | --- | --- |
| I liked participating in the training | 13 | 7 | 6 |
| The training was interesting (because I learnt new things) | 7 | 4 | 3 |
| Some tasks were easy, others were difficult. Not too easy nor too difficult | 9 | 5 | 4 |
| I found that the tasks were too easy, which made it hard for me to motivate myself to go to the training | 1 | 0 | 1 |
| I am not sure whether I would recommend the training with brain stimulation | 6 | 3 | 3 |
| I would recommend the training without brain stimulation | 1 | 0 | 1 |
| I would recommend the training with brain stimulation | 7 | 5 | 2 |
| I would recommend the training to others (CR or combined) (to some people) | 15 | 9 | 6 |
| I would not recommend the training ot others (CR or combined) | 2 | 1 | 1 |
| I liked that the training was personalized | 1 | 0 | 1 |
| I experienced a small discomfort by the tDCS, but I did not mind the brain stimulation. | 6 | 3 | 3 |
| The tDCS was a large burden. | 2 | 1 | 1 |
| It was easy to motivate myself to go to the training | 10 | 6 | 4 |
| Sometimes I found it difficult to motivate myself to go to the training | 4 | 2 | 2 |
| The training was too intense (too many sessions per week) | 4 | 1 | 3 |
| The training intensity was good (some said: it was intense, but not too intense) | 12 | 8 | 4 |
| Lack of understanding the goals of intervention (CIRCuiTS) | 9 | 4 | 5 |
| The goal of the intervention is to improve cognitive functioning | 6 | 2 | 4 |
| Understanding the goals of intervention (accepted: everything regarding goals to improve daily functioning/personal goals) | 7 | 5 | 2 |
| The goal of the intervention is to improve everyday functioning | 4 | 2 | 2 |
| Can explain how the intervention works (how the training can lead to improvements in everyday life) | 3 | 1 | 2 |
| Lack of understanding how the intervention works | 13 | 7 | 6 |
| Understands strategies: explains how strategies can help in everyday life | 1 | 0 | 1 |
| Understands the purpose of strategies: strategies help to do things easier in everyday life (no explanation how) | 1 | 0 | 1 |
| Does not understand strategies | 11 | 5 | 6 |
| Only names 1-2 specific strategies without explanation | 6 | 4 | 2 |
| I cannot remember (the purpose of) the strategies | 4 | 0 | 4 |
| It is difficult to explain (the purpose of (the strategies) | 1 | 1 | 0 |
| Links strategies to helping perform better in training tasks | 5 | 5 | 0 |
| I had to cancel many appointments to be able to take part in the training | 1 | 0 | 1 |
| I did not have to give up anything to engage in the training | 17 | 10 | 7 |
| I think that I would have observed more improvements if I would have continued the training for a longer time | 2 | 2 | 0 |
| More insight into my thinking skills (strengths and weaknesses) | 6 | 3 | 3 |
| I improved my thinking skills | 12 | 7 | 5 |
| I did not observe improved thinking skills | 2 | 1 | 1 |
| I did not observe improvements in my everyday life | 3 | 3 | 0 |
| My thinking skills improved less than I hoped for | 1 | 1 | 0 |
| I observed improvements in my everyday functioning | 14 | 7 | 7 |
| I believe that the brain stimulation had additive value | 4 | 2 | 2 |
| I am not sure whether the brain stimulation added something to the training | 12 | 6 | 6 |
| The strategies I learnt in the training are useful in my everyday life/ help me to do things easier in my everyday life | 7 | 4 | 3 |
| I feel like the improvements are disappearing now that I stopped the training | 2 | 1 | 1 |
| I believe that I can perform the tasks in the training (independently) | 13 | 8 | 5 |
| I believe that I need help to perform the tasks in the training | 3 | 2 | 1 |
| I improved my computer skills | 2 | 0 | 2 |
| I liked that the training was on the computer | 2 | 2 | 0 |
| It is a pity that the training stopped (would have liked to continue) | 4 | 3 | 1 |
| Baat het niet dan schadt het niet ("There is no harm in trying / can't hurt to try") | 3 | 3 | 0 |
| I liked my therapist/I liked the training with my therapist | 15 | 9 | 6 |
| I liked that the therapist was clear/explained the tasks clearly | 8 | 5 | 3 |
| I feel like the training would have been less useful without my therapist | 3 | 2 | 1 |
| There was nothing that I did not like about my therapist | 4 | 2 | 2 |
| I liked that the therapist helped me (with difficult tasks/to get started with tasks)/supported me | 7 | 5 | 2 |
| The therapist made me feel comfortable/safe/gave me self-confidence. | 5 | 2 | 3 |
| I did not have goals during the training/I cannot remember any goals | 3 | 2 | 1 |
| I set goals together with my therapist | 9 | 5 | 4 |
| I set the goals myself | 3 | 2 | 1 |
| I don't remember how the goals were set | 3 | 2 | 1 |
| The goals were important to me | 8 | 3 | 5 |
| The goals were not really in my mind during the training | 1 | 1 | 0 |
| I was thinking about the goals during the training | 1 | 1 | 0 |
| I worked on goals (but does not mention which goals) | 3 | 1 | 2 |
| Mentions goals that were not discussed with the therapist | 1 | 0 | 1 |
| Had goals and can mention the goals: | 11 | 7 | 4 |
| Improving concentration | 7 | 4 | 3 |
| Improving memory | 6 | 3 | 3 |
| Improving planning skills | 1 | 1 | 0 |
| Improving social skills | 1 | 1 | 0 |
| Improving computer skills | 2 | 0 | 2 |
| Specific personal goal, related to daily functioning | 3 | 2 | 1 |
| Taking more rest/being less impulsive | 1 | 1 | 0 |
| Increase self-confidence | 1 | 1 | 0 |

## Table S6. Standardized effect sizes [95% CI] for the main analyses

| Parameter | LSP | NOSCA | Global Cognition | CFQ | SNS |
| --- | --- | --- | --- | --- | --- |
| *Fixed effects* |  |  |  |  |  |
| Intercept | -0.02 [-0.36, 0.32] | 0.03 [-0.28, 0.33] | -0.00 [-0.39, 0.37] | -0.04 [-0.41, 0.32] | -0.04 [-0.39, 0.31] |
| T1 vs. T0 (TAU)^a^ | -0.20 [-0.37, -0.03] | … | … | … | … |
| T1 vs. T2 (CR) | 0.10 [-0.07, 0.27] | 0.05 [-0.15, 0.25] | 0.15 [0.09, 0.22] | -0.03 [-0.15, 0.09] | -0.09 [-0.25, 0.06] |
| T1 vs. T3 | 0.06 [-0.11, 0.23] | 0.00 [-0.20, 0.20] | 0.16 [0.09, 0.24] | -0.06 [-0.18, 0.06] | -0.21 [-0.38, -0.05] |

Note. CFQ = Cognitive Failure Questionnaire, CR = cognitive remediation, LSP = Life Skills Profile, NOSCA = Nurses Observation Scale for Cognitive Abilities, SNS = Self-Report of Negative Symptoms, TAU = treatment as usual. For the LSP, NOSCA and global cognition, a higher score represents a more favorable outcome. For the CFQ and SNS, a lower score represents a more favorable outcome.

^a^… = effect appeared not to be significant (fixed effect) or did not increase the model fit (random fit) and was therefore removed from the model. For each outcome measure, the standardized effect sizes of the model with the best model fit according to the AIC is presented.

## Table S6. Standardized effect sizes [95% CI] for the main analyses including tDCS

| Parameter | LSP | NOSCA | Global Cognition | CFQ | SNS |
| --- | --- | --- | --- | --- | --- |
| *Fixed effects* |  |  |  |  |  |
| Intercept | 0.08 [-0.43, 0.58] | 0.04 [-0.44, 0.51] | -0.21 [-0.74, 0.32] | 0.00 [-0.53, 0.54] | 0.22 [-0.30, 0.75] |
| T1 vs. T0 (TAU)^a^ | -0.20 [-0.36, -0.04] | … | … | … | … |
| T1 vs. T2 (CR) | 0.10 [-0.06, 0.25] | 0.05 [-0.15, 0.25] | **0.16 [0.09, 0.22]** | -0.03 [-0.15, 0.09] | -0.10 [-0.25, 0.05] |
| T1 vs. T3 | 0.03 [-0.15, 0.22] | -0.00 [-0.22, 0.21] | **0.16 [0.09, 0.24]** | -0.06 [-0.18, 0.07] | -0.22 [-0.40, -0.04] |
| tDCS | -0.19 [-0.86, 0.48] | -0.02 [-0.64, 0.60] | 0.37 [-0.34, 1.07] | -0.07 [-0.79, 0.64] | -0.40 [-1.09, 0.29] |

Note. CFQ = Cognitive Failure Questionnaire, CR = cognitive remediation, LSP = Life Skills Profile, NOSCA = Nurses Observation Scale for Cognitive Abilities, SNS = Self-Report of Negative Symptoms, TAU = treatment as usual, tDCS = transcranial direct current stimulation. For the LSP, NOSCA and global cognition, a higher score represents a more favorable outcome. For the CFQ and SNS, a lower score represents a more favorable outcome.

^a^… = effect appeared not to be significant (fixed effect) or did not increase the model fit (random fit) and was therefore removed from the model. For each outcome measure, the standardized effect sizes of the model with the best model fit according to the AIC is presented.

## Table S7. Baseline characteristics completer vs. non-completer

| Baseline Characteristic | Completer  (*n* = 15) | Non-completer  (*n* = 9) | *p* |
| --- | --- | --- | --- |
| Age | 30 (7.4) | 53.3 (14.0) | **.001** |
| Female | 5 | 4 | .913 |
| *Level of education^a^* |  |  | **.017** |
| low | 1 | 4 |  |
| middle | 4 | 4 |  |
| high | 10 | 1 |  |
| *Primary diagnosis* |  |  | .214 |
| Schizophrenia | 3 | 4 |  |
| Autism spectrum disorder | 3 | 1 |  |
| Schizoaffective disorder | 3 | 0 |  |
| Bipolar disorder | 2 | 0 |  |
| Major depressive disorder | 1 | 1 |  |
| Psychosis NOS | 2 | 0 |  |
| Borderline personality disorder | 0 | 1 |  |
| PTSD | 0 | 1 |  |
| Body dysmorphic disorder | 1 | 0 |  |
| Obsessive compulsive disorder | 0 | 1 |  |
| Illness duration | 15.8 (9.6) | 30.8 (12.8) | **.016** |
| Age of onset | 16.6 (6.4) | 22.4 (6.6) | .070 |
| PANSS positive | 10.7 (4) | 14.8 (8.6) | .246 |
| PANSS negative | 13 (6.7) | 18 (8.9) | .211 |
| PANSS general | 28.4 (6.7) | 33.9 (16.5) | .402 |
| PANSS total | 52.1 (13) | 66.6 (33.4) | .276 |
| LSP total | 118.8 (11.9) | 120.8 (16.2) | .742 |
| NOSCA total | 16.9 (1.5) | 16.9 (2) | .979 |
| SNS total | 19.3 (6.1) | 15.5 (7.3) | .240 |
| CFQ total | 37.1 (14.4) | 36.1 (15.5) | .885 |
| Neuropsychological composite | 0.1 (0.9) | -0.5 (0.7) | .073 |

*Note*. CFQ = Cognitive Failure Questionnaire, CR = cognitive remediation, LSP = Life Skills Profile, NOS = not otherwise specified, NOSCA = Nurses Observation Scale of Cognitive Abilities, PANSS = Positive And Negative Syndrome Scale, PTSD = post-traumatic stress disorder, SNS = Self-report of Negative Symptoms, tDCS = transcranial direct current stimulation.

^a^The level of education is categorized using the Dutch Verhage scale (1964)

## Table S8. Baseline characteristics comparison between participants with prolonged waiting periods due to COVID-19 and other participants

| Baseline Characteristic | Prolonged waiting period (n = 7) | Planned waiting period (n = 17) | *p* |
| --- | --- | --- | --- |
| Age | 45.9 (13.0) | 35.8 (15.6) | **.049** |
| Female | 3 (42.8%) | 6 (35.3%) | .999 |
| *Level of education^a^* |  |  | .550 |
| low | 2 (28.6%) | 3 (17.6%) |  |
| middle | 3 (42.8%) | 5 (29.4%) |  |
| high | 2 (28.6%) | 9 (52.9%) |  |
| *Primary diagnosis* |  |  | .329 |
| Schizophrenia | 2 (28.6%) | 5 (29.4%) |  |
| Autism spectrum disorder | 1 (14.3%) | 3 (17.6%) |  |
| Schizoaffective disorder | 1 (14.3%) | 2 (11.8%) |  |
| Bipolar disorder | 1 (14.3%) | 1 (5.9%) |  |
| Major depressive disorder | 1 (14.3%) | 1 (5.9%) |  |
| Psychosis NOS | 0 | 2 (11.8%) |  |
| Borderline personality disorder | 0 | 1 (5.9%) |  |
| PTSD | 0 | 1 (5.9%) |  |
| Body dysmorphic disorder | 0 | 1 (5.9%) |  |
| Obsessive compulsive disorder | 1 (14.3%) | 0 |  |
| Illness duration | 26.3 (14.2) | 17.5 (7.8) | .204 |
| Age of onset | 21.4 (4.3) | 17.5 (7.8) | .130 |
| PANSS positive | 17.5 (9.0) | 10 (3.0) | **.038** |
| PANSS negative | 13.8 (6.0) | 18.2 (10.9) | .422 |
| PANSS general | 38.5 (18.0) | 27.0 (5.2) | **.043** |
| PANSS total | 74.2 (37.2) | 50.8 (10.1) | .092 |
| LSP | 112 (12.5) | 123 (12.7) | .099 |
| NOSCA | 16.4 (1.9) | 17.1 (1.6) | .431 |
| Global Cognition | -0.49 (0.59) | 0.03 (0.62) | .166 |
| CFQ | 40.7 (14.9) | 34.8 (14.5) | .323 |
| SNS | 19 (8.1) | 17.3 (6.0) | .940 |
| tDCS |  |  | .793 |
| active | 3 (42.8%) | 10 (58.8%) |  |
| sham | 4 (57.2%) | 7 (41.2%) |  |

*Note*. CFQ = Cognitive Failure Questionnaire, CR = cognitive remediation, LSP = Life Skills Profile, NOS = not otherwise specified, NOSCA = Nurses Observation Scale of Cognitive Abilities, PANSS = Positive And Negative Syndrome Scale, PTSD = post-traumatic stress disorder, SNS = Self-report of Negative Symptoms, tDCS = transcranial direct current stimulation. Given the group size differences and small sample sizes, P-values for continuous outcomes were derived from Mann-Whitney U tests, comparing the distributions between two independent groups. P-values for categorical outcomes were derived from Chi-Square tests.

^a^The level of education is categorized using the Dutch Verhage scale (1964)

## Table S9. Comparison of changes during the waiting period between participants with prolonged waiting periods due to COVID-19 and other participants

|  | Prolonged waiting period | | | Planned waiting period | | | Group comparison | |
| --- | --- | --- | --- | --- | --- | --- | --- | --- |
| Parameter | T0  Mean (SD) | T1  Mean (SD) | Mean difference  Mean (SD) | T0  Mean (SD) | T1  Mean (SD) | Mean difference  Mean (SD) | W | p |
| LSP | 112 (12.5) | 125 (10.4) | 10.7 (8.5) | 123 (12.7) | 126 (9.0) | 2.0 (12.3) | 70.5 | .105 |
| NOSCA | 16.4 (1.9) | 17.2 (1.6) | 0.0 (1.7) | 17.1 (1.6) | 17.4 (1.6) | 0.3 (1.9) | 36 | .780 |
| Global Cognition | -0.49 (0.59) | -0.65 (0.61) | -0.16 (0.22) | 0.03 (0.62) | 0.04 (0.67) | 0.01 (0.28) | 40 | .234 |
| CFQ | 40.7 (14.9) | 47 (15.2) | 6.3 (10.8) | 34.8 (14.5) | 28.7 (14.2) | -6.2 (10.1) | 78 | .033* |
| SNS | 19 (8.1) | 20.5 (7.5) | 1.5 (7.3) | 17.3 (6.0) | 18.8 (7.7) | 1.3 (5.3) | 45 | 1 |

Note. CFQ = Cognitive Failure Questionnaire, CR = cognitive remediation, LSP = Life Skills Profile, NOSCA = Nurses Observation Scale for Cognitive Abilities, SNS = Self-Report of Negative Symptoms, TAU = treatment as usual, tDCS = transcranial direct current stimulation. For the LSP, NOSCA and global cognition, a higher score represents a more favorable outcome. For the CFQ and SNS, a lower score represents a more favorable outcome. P-values were derived from Mann-Whitney U tests, comparing the mean differences between two independent groups.
**P* < .05

## References

Broadbent, D. E., Cooper, P. F., FitzGerald, P., & Parkes, K. R. (1982). The Cognitive Failures Questionnaire (CFQ) and its correlates. *British Journal of Clinical Psychology*, *21*(1), 1–16. https://doi.org/10.1111/j.2044-8260.1982.tb01421.x

Dollfus, S., Delouche, C., Hervochon, C., Mach, C., Bourgeois, V., Rotharmel, M., Trehout, M., Vandevelde, A., Guillin, O., & Morello, R. (2019). Specificity and sensitivity of the Self-assessment of Negative Symptoms (SNS) in patients with schizophrenia. *Schizophrenia Research*, *211*, 51–55. https://doi.org/10.1016/j.schres.2019.07.012

Kay, S. R., Fiszbein, A., & Opler, L. A. (1987). The positive and negative syndrome scale (PANSS) for schizophrenia. *Schizophrenia Bulletin*, *13*, 261–276. https://doi.org/10.1093/schbul/13.2.261

Nelson, H. E. (1976). A Modified Card Sorting Test Sensitive to Frontal Lobe Defects. *Cortex*, *12*, 313–324. https://doi.org/10.1016/s0010-9452(76)80035-4

Osterrieth, P. A. (1944). Le test de copie d’une figure complexe; contribution à l’étude de la perception et de la mémoire. [Test of copying a complex figure; contribution to the study of perception and memory.]. *Archives de Psychologie*, *30*, 206–356.

Persoon, A., Schoonhoven, L., Melis, R. J. F., Achterberg, T. V., Kessels, R. P. C., & Rikkert, M. G. M. O. (2012). Validation of the NOSCA - nurses’ observation scale of cognitive abilities. *Journal of Clinical Nursing*, *21*, 3025–3036. https://doi.org/10.1111/j.1365-2702.2012.04129.x

Ponds, R., Boxtel, M. P. J. V., & Jolles, J. (2006). De “Cognitive Failure Questionnaire” als maat voor subjectief cognitief functioneren [The ‘Cognitive Failure Questionnaire’ a measure of subjective cognitive functioning]. *Tijdschrift Voor Neuropsychologie*, *2*, 37–45. https://www.researchgate.net/publication/286212206

Rey, A. (1941). L’examen psychologique dans les cas d’encéphalopathie traumatique. *Archives de Psychologie*, *28*, 215–285.

Rosen, A., Hadzi-Pavlovic, D., & Parker, G. (1989). The Life Skills Profile: A measure assessing function and disability in schizophrenia. *Schizophrenia Bulletin*, *15*(2), 325–337. https://doi.org/10.1093/schbul/15.2.325

Saan, R., & Deelman, B. (1986). Nieuwe 15-woorden test A en B (15WTA en 15WTB). In ["A Bouma", "J Mulder", & "J Lindeboom"] (Eds.), *Neuro-psychologische diagnostiek : Handboek* (pp. 13–28). Swets & Zeitlinger.

Saturnino, G. B., Siebner, H. R., Thielscher, A., & Madsen, K. H. (2019). Accessibility of cortical regions to focal TES: Dependence on spatial position, safety, and practical constraints. *NeuroImage*, *203*, 116183. https://doi.org/10.1016/j.neuroimage.2019.116183

Schmand, B., Groenink, S. C., & Dungen, M. den. (2008). Letterfluency: psychometrische eigenschappen en Nederlandse normen. *Tijdschrift Voor Gerontologie En Geriatrie*, *39*, 64–74. https://doi.org/10.1007/bf03078128

Stroop, J. R. (1935). Studies of interference in serial verbal reactions. *Journal of Experimental Psychology*, *18*, 643–662. https://doi.org/10.1037/h0054651

Wechsler, D. (2008). *Wechsler adult intelligence scale - Fourth Edition (WAIS-IV)*. NCS Pearson.
